# ECGA: A web server to explore and analyze extrachromosomal gene in cancer

**DOI:** 10.1016/j.csbj.2024.11.009

**Published:** 2024-11-05

**Authors:** Nan Zhou, Li Peng, Zhiyu Zhang, Qiqi Luo, Huiran Sun, Jinku Bao, Yuping Ning, Xiaoqing Yuan

**Affiliations:** a Research Center, The Affiliated Brain Hospital, Guangzhou Medical University, Guangzhou 510370, China; b Medical Research Center, Guangdong Provincial Key Laboratory of Malignant Tumor Epigenetics and Gene Regulation, Guangdong-Hong Kong Joint Laboratory for RNA Medicine, Medical Research Center, Sun Yat-Sen Memorial Hospital, Sun Yat-Sen University, Guangzhou 510120, China; c College of Life Sciences, Sichuan University, Chengdu 610064, China; d Guangdong Engineering Technology Research Center for Translational Medicine of Mental Disorders, Guangzhou 510370, China; e Key Laboratory of Neurogenetics and Channelopathies of Guangdong Province and the Ministry of Education of China, Guangzhou Medical University, Guangzhou 510000, China; f Guangdong Provincial Key Laboratory of Cancer Pathogenesis and Precision Diagnosis and Treatment, Shenshan Medical Center, Sun Yat-sen Memorial Hospital, Sun Yat-sen University, Shanwei 516621, China

**Keywords:** Extrachromosomal DNA, ecDNA gene, Web server, Analysis

## Abstract

Circular extrachromosomal DNA (ecDNA) plays a crucial role in the onset, progression, and evolution of many types of cancers, with dysregulated gene expression driven by ecDNA as a key mechanism. Although database resources for ecDNA are now available, a sophisticated web application dedicated to ecDNA gene analysis remains absent. Therefore, we developed ecDNA gene analyzer (ECGA). ECGA catalogues 23,274 unique ecDNA genes of 27 cancers across 27 tissues. ECGA also offers five specialized analysis tools: (1) ‘Venn analysis’ looks for overlaps between a given gene list and ecDNA genes; (2) ‘Enrichment analysis’ performs over-representation analysis and gene set enrichment analysis of input gene list within predefined ecDNA gene sets; (3) ‘Target discovery’ identifies upregulated ecDNA genes as targets by comparing with reference expression in normal samples; (4) ‘DE analysis’ finds differentially expressed ecDNA genes; (5) ‘Signature discovery’ discerns ecDNA gene signatures capable of classifying samples into phenotypic groups, and it is accompanied by ‘Signature validation’ for model test on unseen data. In summary, ECGA emerges as an indispensable platform in cancer genetics, bridging gaps in basic research, medical reporting, and pharmaceutical development, and propelling ecDNA research forward. ECGA is freely available at https://www.zhounan.org/ecga/.

## Highlights

• ECGA provides a comprehensive catalogue of ecDNA genes in cancer.
• ECGA contains 23,274 unique ecDNA genes of 27 cancers.
• ECGA is the first web server focusing on ecDNA gene analysis.
• ECGA provides a user-friendly interface to simplify analysis task.
• ECGA addresses a variety of analysis needs in ecDNA gene research.

## Graphical Abstract

**Figure:**
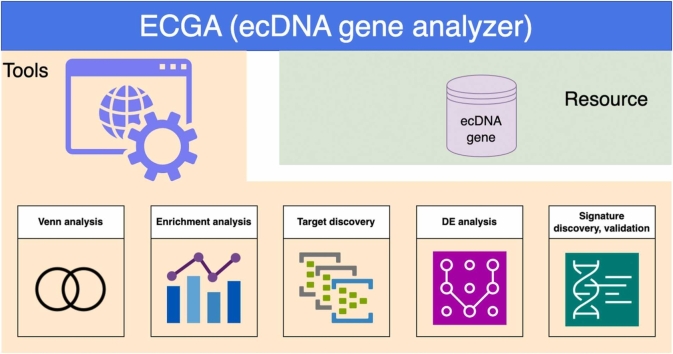

## 1 Introduction

Extrachromosomal DNA (ecDNA) is a type of circular DNA element that encompasses the full spectrum of large, gene-containing extrachromosomal particles of DNA, including both double minute and single body forms and forms lacking a centromere or a telomere [1]. The first description of ecDNA dates back to 1965 when double minutes were observed in neuroblastoma cell lines [2]. An increasing number of reports have demonstrated that ecDNAs are prevalent in cancer genomes and have emerged as a crucial oncogenic driver [3], [4], [5], [6].

Numerous alterations in DNA sequence underlie the development of every neoplasm, and hence a central aim of cancer research has been to identify the mutated genes that are causally implicated in oncogenesis [7]. As one of the most common molecular alterations in cancer, oncogene amplification plays a central role in tumorigenesis by providing cancer cells with selective growth advantages through overexpression of oncogenes and functional elements [8], [9]. Among many of the genomic events that enable to lead up to the very high level of gene expression, gene-carrying ecDNA is a potent and frequent mechanism by which genes are amplified and that such ecDNA can foment increased intratumoural genetic heterogeneity, owing to its circular structure and the lack of centromeres [10], [11].

The concept that genes can reside on ecDNA is not new. As early in the 1980s, Kohl and colleagues demonstrated that sequences resembling MYCN could be mapped to double minutes in the IMR-32 neuroblastoma cell line [12]. Recent re-evaluation of ecDNA in large-scale DNA sequencing data revealed that oncogene amplification on ecDNA is a frequent event in cancer and promotes tumor heterogeneity [13]. After analyzing 117 cancer genomes and 2572 metaphase cells, Turner and colleagues discovered that ecDNAs exist in nearly half of human cancers and that oncogenes on ecDNAs are amplified most commonly [11]. Wu and colleagues found that ecDNA promotes accessible chromatin and that oncogenes encoded on ecDNA are among the most highly expressed genes in the transcriptome in tumors [6]. By analyzing whole-genome sequencing data of 3212 cancer patients, Kim and colleagues found that the most common recurrent oncogene amplifications arose on ecDNA and that patients whose cancers carried ecDNA had significantly shorter survival than patients whose cancers were not driven by ecDNA-based oncogene amplification [14].

To support ecDNA research, a few web resources have been created. Our previous work eccDNAdb is the first database for ecDNAs in cancer [15]. TeCD provides extrachromosomal circular DNA (eccDNA) found in animal, plants, and fungi [16]. EccBase provides eccDNAs from healthy and tumor samples in human and mouse [17]. The eccDNA Atlas database provides eccDNAs for health and diseases across multiple specie [18]. CircleBase contains human eccDNAs which are gleaned from published papers [19]. These database resources specifically focus on ecDNA, not the cargo genes (referred to here as ‘ecDNA gene’), let alone featured analysis, which is not proportional to the importance of ecDNA gene in cancer.

Currently there is no tools providing a freely available user-friendly web tool to explore and analyze ecDNA genes in cancer. To bridge the gap, we created ECGA, for easy exploration of a comprehensive list of ecDNA genes in cancer and specialized analyses. The ecDNA genes in ECGA were identified from 27 cancers across 27 tissues. Five dedicated tools were created to cover a wide range of analysis requests which are common in daily oncology research work. ECGA does not require registration or login for access, and it is freely available for research use from multiple devices, screen sizes and browsers.

## 2 Methods and materials

**Fig. 1:**
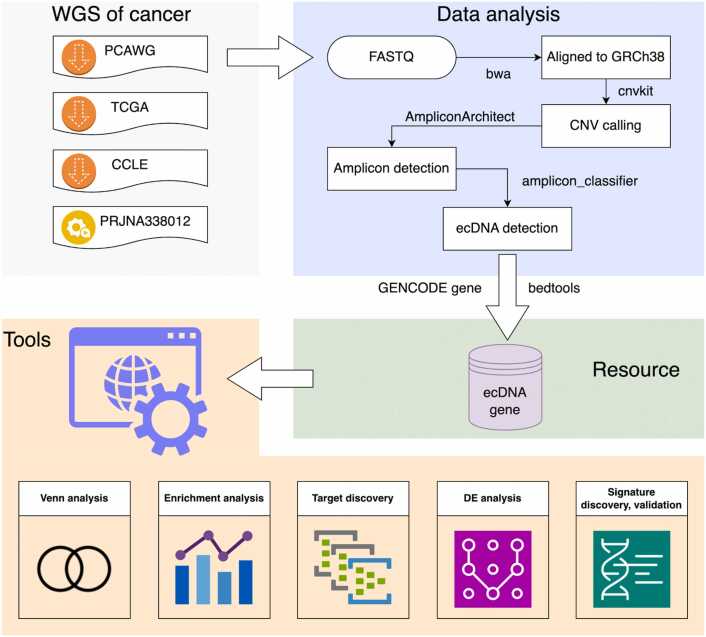
Schematic presentation of the workflow of ECGA. The WGS data of cancers were downloaded from public repositories. The data analysis pipeline was then performed to analyze and detect ecDNA. Intersections between genes and ecDNAs were analyzed to identify ecDNA genes, which subsequently formed the ecDNA gene resource. Finally, a collection of tools dedicated to ecDNA gene analyses were created. This diagram was created with icons from draw.io.

ECGA consists of two parts: (1) an ecDNA gene resource which contains a comprehensive catalogue of ecDNA genes identified in cancer genomes by analyzing whole genome sequencing data, and (2) a suite of tools dedicated to ecDNA gene analysis. The overall workflow is shown in Fig. 1. The description of parameters across tools is presented in Supplementary Table 1.

### 2.1 Whole genome sequencing data collection

To generate a catalogue of ecDNA genes for cancers as comprehensive as possible, a wide range of cancers of different genetic backgrounds should be examined. Considering both the data availability and sample variety, whole genome sequencing (WGS) data derived from a large population of tissues and cell lines of different cancers is an ideal start point. Therefore, WGS data from Pan-Cancer Analysis of Whole Genomes (PCAWG), The Cancer Genome Atlas (TCGA), Cancer Cell Line Encyclopedia (CCLE), and NCBI’s Bioproject PRJNA338012 were used [11], [20], [21].

The naming for phenotypic attributes of samples can vary between data sets. When inconsistence appears, tissue names were standardized as the primary tissue name (e.g., lung), and diseases were named by the tissue name followed by the word cancer (e.g., lung cancer).

### 2.2 ecDNA gene identification

Since the methodological advancement in ecDNA analysis, here we used a similar but slightly different strategy of our previous work to discover ecDNA in cancer genomes [15]. Sequencing reads were aligned to the human reference genome GRCh38 using the bwa mem method [22]. Aligned reads were sorted and indexed using samtools [23]. A pooled reference of per-bin copy number estimates from normal samples was created, and then copy number variation (CNV) in tumor was inferred using CNVkit [24]. CNVs were filtered and merged into seed intervals via PrepareAA. Afterwards, amplicons were identified by AmpliconArchitect (AA) [25]. To detect ecDNA positive amplicons, AA outputs were subsequently analyzed by AmpliconClassifer to obtain ecDNAs and their genomic coordinates [26]. Default settings were applied to the software except where noted.

To identify ecDNA genes, human gene annotation data in the GENCODE database (v44 for GRCh38 and v46 for GRCh37) was downloaded [27]. The bedtools utilities were used to map reference gene features from GENCODE onto the genomic intervals of ecDNAs to find overlaps between the gene and ecDNA. For a gene having intersections with ecDNA, it was selected as an ecDNA gene, and the overlapping ratio was calculated by the number of base pairs of intersection divided by the gene length. To estimate the merit of a gene being an ecDNA gene, the ratio of overlap per gene–ecDNA pair was defined as ecDNA gene score and the total number of ecDNAs the gene overlaps was defined as ecDNA hits.

Regarding to the four collected data sets, we only analyzed the PRJNA338012 WGS data. Others have been analyzed by Kim and colleagues, and the results in ready-to-use format have been shared through AmpliconRepository (https://dev.ampliconrepository.org/) [25], [28]. We downloaded CCLE results in June 2023 and PCAWG and TCGA results in October 2024, respectively. With these downloaded data, we extracted ecDNA genes and then calculated ecDNA gene scores and ecDNA hits ourselves. Of note, both parts of ecDNA genes were identified using compatible software and pipelines, so we merged them into a uniformly formatted data set and then used it as the ECGA’s ecDNA gene resource and subsequently developed a series of analysis tools.

### 2.3 External resource integration

To explore ecDNA gene expression profiles in multi-omics data derived from a variety of biological contexts, resources from GeneRanger and UCSC Xena browser were incorporated [29], [30]. For the estimation of tumor survival on ecDNA gene, data from FerrDb were integrated [31].

### 2.4 Analysis tool development

There are five dedicated tools for ecDNA gene analysis. ‘Venn analysis’ was developed using the venn2 and venn2_unweighted Python packages. ‘Enrichment analysis’ was developed using gseapy and it can be used for enrichment based on over-representation analysis (ORA) and gene set enrichment analysis (GSEA) [32]. ‘Target discovery’ was developed with the computation service provided by TargetRanger [29]. ‘DE analysis’ was developed using limma and DESeq2 [33], [34]. ‘Signature discovery’ and its optional auxiliary ‘Signature validation’ tools were developed using the PyCaret python library (https://pycaret.gitbook.io/docs/).

### 2.5 Website development and deployment

The ECGA website was developed using cutting-edge client-side techniques, such as HTML5, CSS3 and JavaScript. The jQuery (https://jquery.com/) library was used to assist JavaScript coding. Bootstrap (https://getbootstrap.com/) was used to streamline the user interface and experience for diverse devices, browsers, and screen sizes. DataTables (https://datatables.net/) was used to create interactive data tables on the web page. Interactive diagrams on the web page were created using plotly (https://plotly.com/). On the server side, Python (https://python.org/) and the Django (https://www.djangoproject.com/) framework were used to build web applications, data were stored in text files or SQLite (https://www.sqlite.org/) whichever is appropriate, analysis scripts were written in Python and R (https://www.r-project.org/), and client-server communications were supported by the Django REST framework (https://www.django-rest-framework.org/) and django-cors-headers (https://pypi.org/project/django-cors-headers/). Finally, the ECGA website was deployed using an Apache2 server in Ubuntu 16.04 on the Amazon Web Services (AWS) cloud.

### 2.6 Data for application cases

The results section will describe the tools and their uses. We will showcase how these tools can be used to re-analyze specific experimental data from published studies, focusing on cases where such re-analysis offers valuable insights. The data sets for application cases were prepared as follows:

(1) OV-TCGA-GTEx: Gene expression (log2-transformed normalized count) and phenotype of ovarian cancer of the UCSC Xena's TCGA TARGET GTEx cohort [30]. TARGET and non-OV samples were removed. TCGA samples were labeled as tumor, whilst GTEx samples were labeled as normal. The data for downstream analysis includes 515 samples and 58,581 genes.
(2) OV-2009: Gene expression (microarray) and phenotype of ovarian cancer of the UCSC Xena's Ovarian Cancer (Etemadmoghadam 2009) cohort [35]. Non-ovary samples were removed. Samples without grade or stage information were also removed. As this data set lacks tumor-normal pairs, samples of grade 1 and type LMP (low-malignant potential) were labeled as normal, and others were labeled as tumor. The data for downstream analysis includes 237 samples and 20,373 genes.

### 2.7 Annotation of ecDNA oncogene

Oncogenes were downloaded from two databases on 22 October, 2024: (1) COSMIC (v100) Cancer Gene Census and (2) ONGene [36], [37]. The COSMIC collection includes 581 oncogenes, and the ONGene collection includes 803 oncogenes. We merged the two collections into a single collection, and the final data set for use in this study includes 1180 unique oncogenes. Finally, ecDNA genes that are also oncogenes were annotated as ecDNA oncogenes.

## 3 Results

### 3.1 ecDNA gene in cancer

The ecDNA gene resource contains 58,800 records in total, which include duplicates because the same ecDNA gene can be identified from different ecDNAs of the same or different samples. Including duplicates may introduce bias into ecDNA gene counts due to intratumor heterogeneity and varying sample sizes. It is intuitive that the bias primarily affects quantitative comparisons of ecDNA genes between samples or cancers. Researchers should exercise caution when conducting such analysis. However, for qualitative analyses, such as identifying the existence of ecDNA genes, the impact of this bias is minimal. Removing duplicates by gene symbol yielded 23,274 unique ecDNA genes, while using gene identifier (i.e., Ensembl gene ID) resulted in 24,128 unique ecDNA genes. Of note, the results presented below in this section are based on ecDNA genes after the removal of duplicates by gene symbol.

**Fig. 2:**
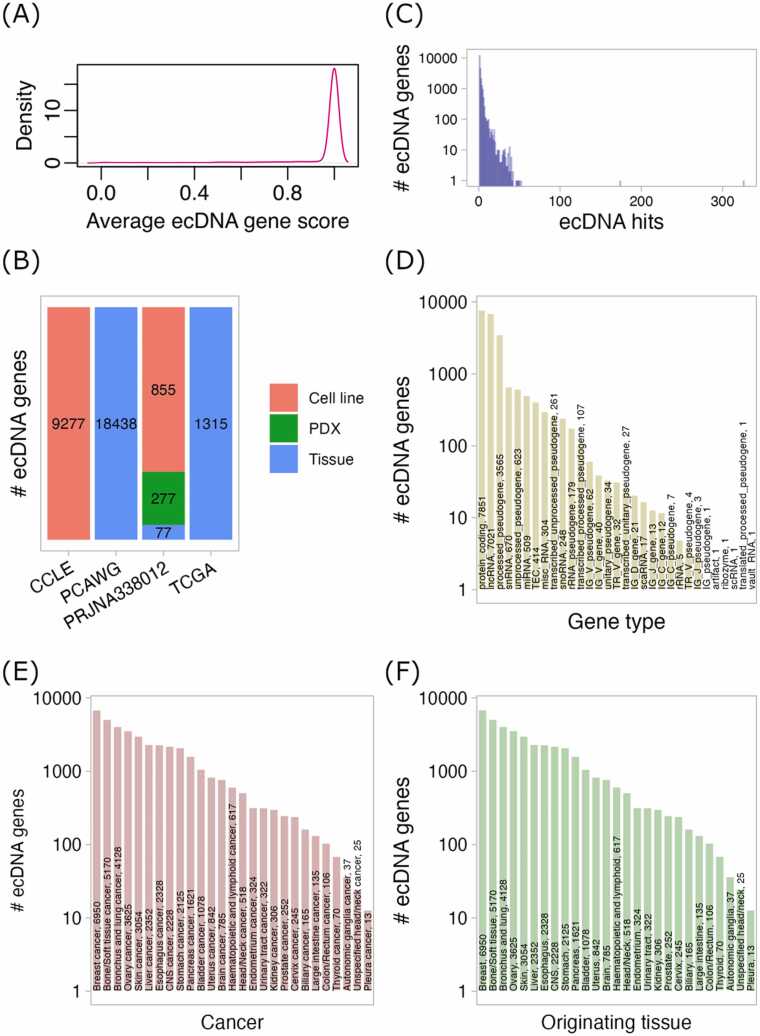
Statistics of ecDNA genes in cancer. (A) The distribution of ecDNA gene scores, averaged for duplicated genes. (B) The number of unique ecDNA genes identified in data sets PCAWG, TCGA, CCLE and PRJNA338012. (C) The number of unique ecDNA genes across ecDNA hits. (D) The distribution of unique ecDNA genes across gene types defined by GENCODE (genes of undefined type are not shown). (E) The distribution of unique ecDNA genes across cancers. (F) The distribution of unique ecDNA genes across tissues of origin.

The majority of the identified ecDNA genes have a high score very close to 1 (Fig. 2A), indicating that full-length genes are contained on ecDNA. As can be seen in Fig. 2B, the number of identified ecDNA genes varies across data sets, with the PCAWG data yielding the highest number and the PRJNA338012 data the lowest (Fig. 2B); cancer tissues yielded the most ecDNA genes, while PDX (patient-derived xenografts) samples yielded the fewest. Nearly 47 % of ecDNA genes are carried on at least two ecDNAs (i.e., ecDNA hits > 1), with 12,406 ecDNA genes residing only on one ecDNA (Fig. 2C). The identified ecDNA genes belong to a variety of types, with protein coding gene, lncRNA, and processed pseudogene as the three most abundant types (Fig. 2D). The largest number of ecDNA genes are from breast cancer, followed by bone/soft tissue cancer, bronchus and lung cancer, ovary cancer, and skin cancer which have > 3000 ecDNA genes (Fig. 2E). Reasonably, similar trend is observed in the distribution of ecDNA genes across tissues of origin (Fig. 2F).

### 3.2 Website overview

**Fig. 3:**
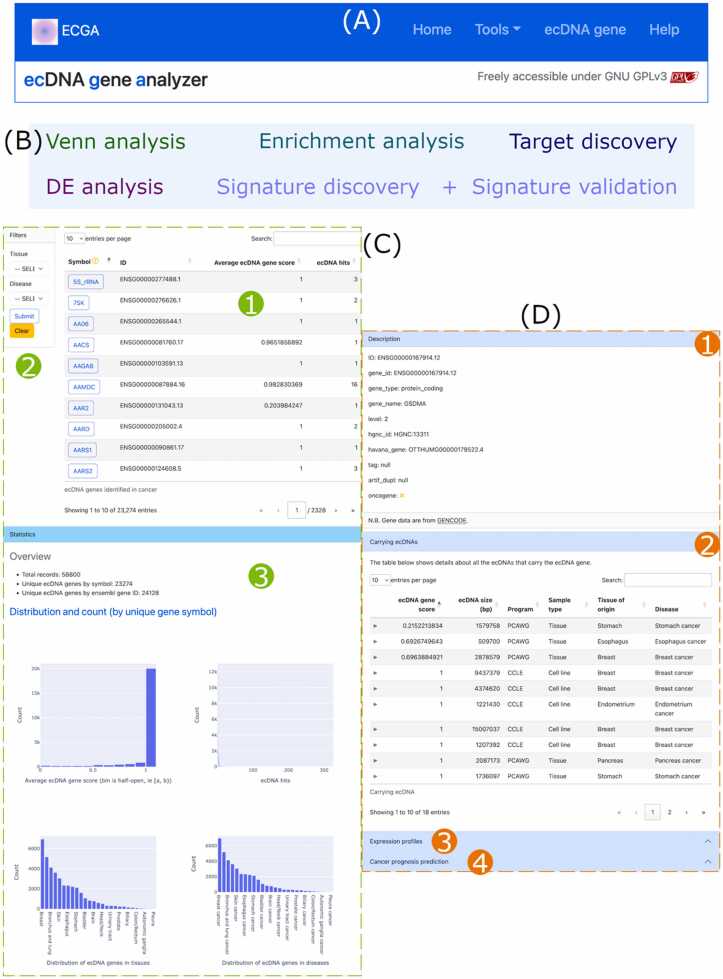
Website overview. (A) Screenshot of the navigation bar. (B) Links to analysis tools under the ‘Tools’ dropdown menu. (C) Screenshot of the ecDNA gene resource page that shows the table (1), filter (2), and statistics (3) of ecDNA genes. (D) Screenshot of an ecDNA gene detail page that shows gene description (1), carrying ecDNAs (2), expression profiles (3), and cancer prognosis prediction (4).

Links to all resources in ECGA are provided in the navigation bar (Fig. 3A). Five analysis tools are available under the ‘Tools’ drop-down menu (Fig. 3B). Of note, ‘Signature validation’ validates ecDNA gene signatures discovered by ‘Signature discovery’. We treat it as part of the ‘Signature discovery’ tool and only provide the link to it when ecDNA gene signature analysis has completed. On the page for the ecDNA gene resource in cancer (Fig. 3C), ecDNA genes are shown in a table (Fig. 3C1), ecDNA genes can be filtered by tissue and disease name using the filter on the left (Fig. 3C2), and statistics of ecDNA genes are also available (Fig. 3C3). Clicking on a gene symbol in the ecDNA gene table will open its detail page (Fig. 3D), where basic description of the gene (Fig. 3D1), gene-carrying ecDNAs (Fig. 3D2), expression profiles (Fig. 3D3), and prognosis prediction of TCGA cancers (Fig. 3D4) are available.

### 3.3 DE analysis

**Fig. 4:**
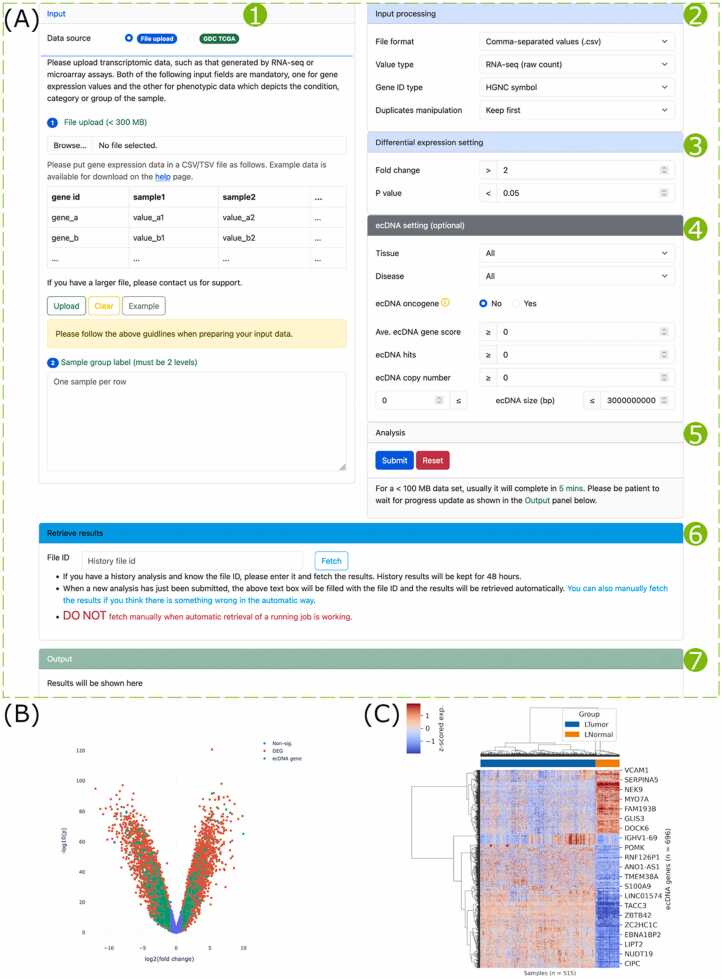
Screenshots and application of the ‘DE analysis’ tool. (A) The tool’s web page showing the data input panel (1), input processing parameters panel (2), differential expression setting panel (3), ecDNA setting panel (4), analysis controller panel (5), result retrieval panel (6), and output panel (7). (B) Differentially expressed ecDNA genes in OV-TCGA-GTEx. (C) Hierarchical clustering of OV-TCGA-GTEx samples by differentially expressed ecDNA genes. Input data description: 58,581 genes × 515 samples. Processing time: 132 s. Non-default parameters: Set ‘Value type’ to ‘RNA-seq (other)’ and ‘Disease’ to ‘Ovary cancer’.

Differential expression analysis of genes in transcriptomics data can be performed using the ‘DE analysis’ tool (Fig. 4A). Users can either upload their gene expression data or choose a TCGA data set which has been pre-processed by us (Fig. 4A1). When all parameters are properly set (Fig. 4A2–4), the analysis can be launched (Fig. 4A5). In the ‘Retrieve results’ panel (Fig. 4A6), history analysis can be manually fetched. In the ‘Output’ panel, analysis progress will be logged, and results will be displayed (Fig. 4A7).

To demonstrate its application, we analyzed the OV-TCGA-GTEx data using the ‘DE analysis’ tool. It discovered 14,692 DEGs in ovary cancer, of which 6910 are upregulated and 7782 are downregulated (Fig. 4B, Supplementary Table 2). Among these DEGs, 696 are ecDNA genes. Hierarchical clustering based on these differentially expressed ecDNA genes successfully separated tumor and normal samples into distinct clusters (Fig. 4C).

### 3.4 Venn analysis

**Fig. 5:**
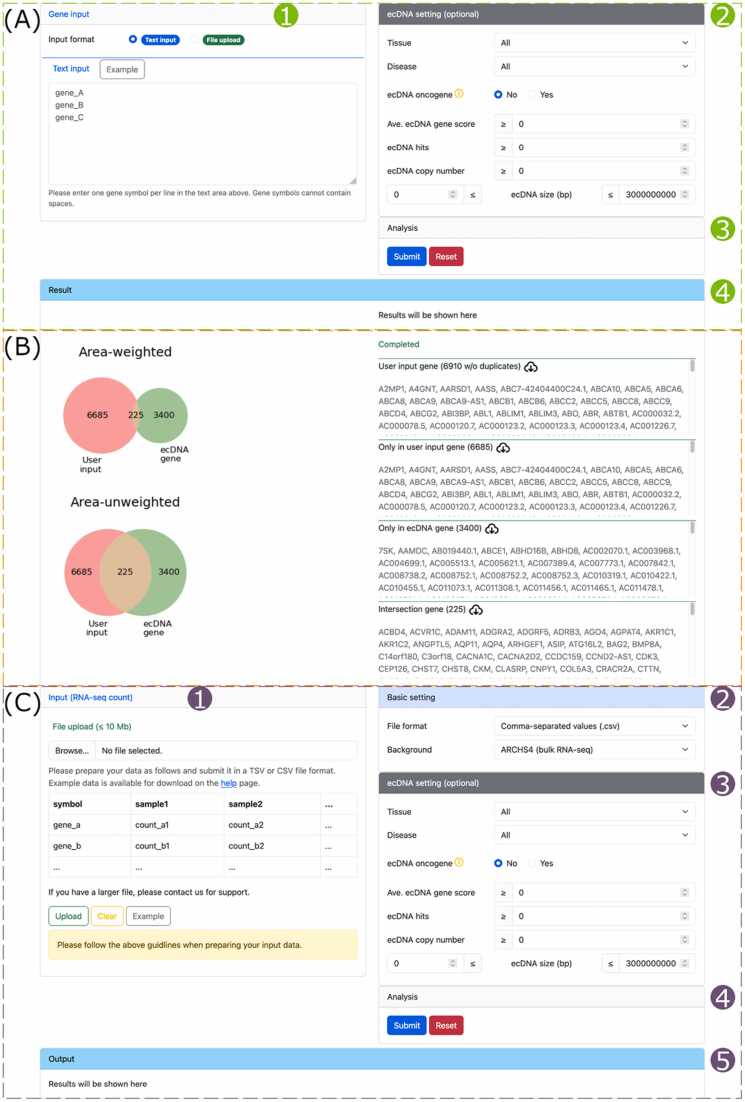
Screenshots and application of the ‘Venn analysis’ and ‘Target discovery’ tools. (A) Web page of the ‘Venn analysis’ tool showing the gene input panel (1), ecDNA setting panel (2), analysis controller panel (3), and output panel (4). (B) Intersections between upregulated DEGs in OV-TCGA-GTEx and ECGA’s ecDNA gene resource. Input data description: 6910 genes. Processing time: 3 s. Non-default parameters: Set ‘Disease’ to ‘Ovary cancer’ (C) Web page of the ‘Target discovery’ tool showing the input panel (1), basic setting panel (2), ecDNA setting panel (3), analysis controller panel (4), and output panel (5).

The ‘Venn analysis’ tool finds ecDNA genes in an input gene list by comparing it with the ecDNA gene resource of ECGA. Users can use the text area to enter genes or upload genes in a file (Fig. 5A1). The ecDNA genes to compare can be filtered using the ‘ecDNA setting’ parameters (Fig. 5A2). After submitting (Fig. 5A3), the results will be shown in the ‘Output’ panel once completed (Fig. 5A4).

We use the aforementioned DEGs of OV-TCGA-GTEx from ‘DE analysis’ results to demonstrate its application. We extracted 6910 upregulated DEGs, entered them into the text area, and then set the disease ‘Ovary cancer’. The results show that 225 DEGs are ecDNA genes, accounting for 6.2 % of all ecDNA genes in ovary cancer (Fig. 5B). Similarly, a previous study found 198 ecDNA genes out of 2188 upregulated DEGs in ovary cancer, making up 9.05 % of all ecDNA genes found in ovarian cancer cell line UACC-1598-4 by the Circle-Seq pipeline [38], [39]. The consistence highlights critical roles of ecDNA genes in ovary cancer, while the subtle variance may come from technical and biological differences.

### 3.5 Target discovery

The ‘Target discovery’ tool finds ecDNA genes highly expressed in a specific biological condition compared to normal human samples, potentially serving as targets for further investigation. The input to ‘Target discovery’ is a file that contains raw RNA-seq count data (Fig. 5C1). After setting parameters (Fig. 5C2–3), the analysis can be launched (Fig. 5C4), and the results will be shown in the output box when completed (Fig. 5C5). Of note, the computation service of this tool is offered by TargetRanger [29].

### 3.6 Enrichment analysis

**Fig. 6:**
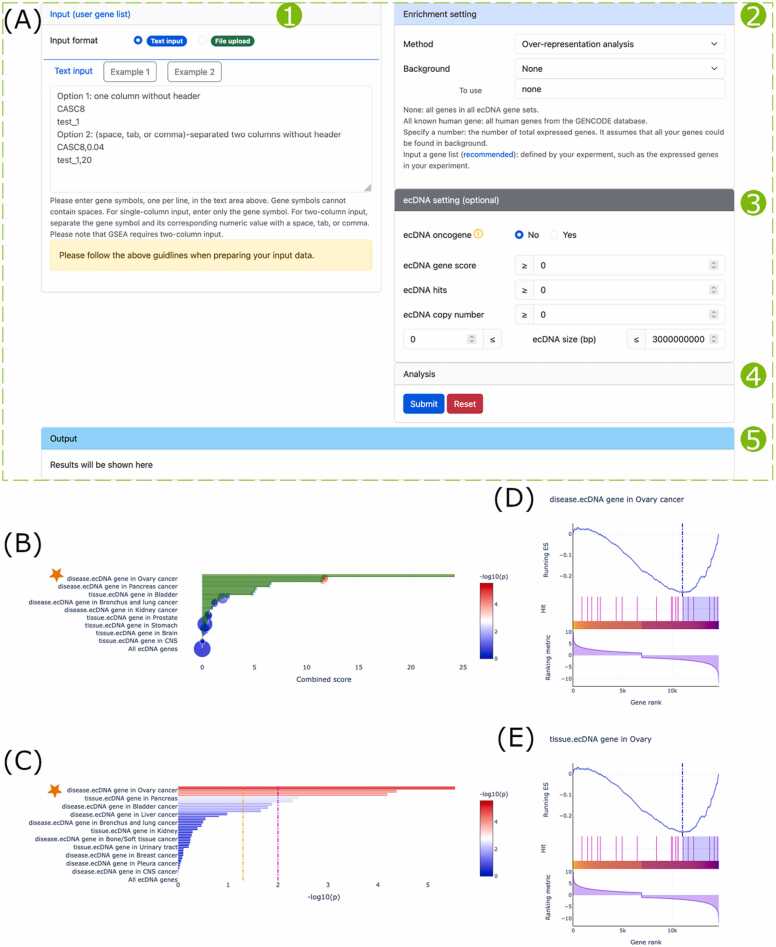
Screenshots and application of the ‘Enrichment analysis’ tool. (A) The tool’s web page showing the input panel (1), enrichment setting panel (2), ecDNA setting panel (3), analysis controller panel (4), and output panel (5). (B–C) ORA-based enrichment analysis results of all DEGs of OV-TCGA-GTEx, with the location of two significantly enriched ovary cancer-related terms indicated by an orange asterisk. (D-E) GSEA-based enrichment analysis results of the two ovary cancer-related terms in (B) and (C). Of note, negative ranking metric values in (D) and (E) denote higher gene expression levels in tumor than in normal. Input data description: 14,692 rows × 2 columns. Processing time: 13 s and 18 s for ORA- and GSEA-based analysis, respectively.

The ‘Enrichment analysis’ tool identifies ecDNA gene sets particularly abundant in a group of genes more than would be expected by chance. A list of genes with or without ranking metrics can be entered in the text area or uploaded as a text file (Fig. 6A1). After setting enrichment method (Fig. 6A2) and ecDNA filters (Fig. 6A3), the enrichment analysis can be launched (Fig. 6A4) and the results will be shown in the output box (Fig. 6A5).

Again, we use the aforementioned DEGs of OV-TCGA-GTEx from ‘DE analysis’ results to demonstrate its application. All DEGs and corresponding log2-transformed fold change values were used as input. The ORA-based enrichment analysis shows that gene sets related to ecDNA genes in ovary cancer are significantly enriched (Fig. 6B and C). GSEA-based enrichment analysis was performed as well, and the results display that the two ecDNA gene sets in ovary cancer are significantly enriched as well (Fig. 6D and E).

### 3.7 Signature discovery and validation

The ‘Signature discovery’ tool discerns ecDNA gene signatures that can be used for diagnosis, prognosis, and drug response predictions. The outlook of this tool is the same as that of ‘DE analysis’ (Fig. 4A).

**Fig. 7:**
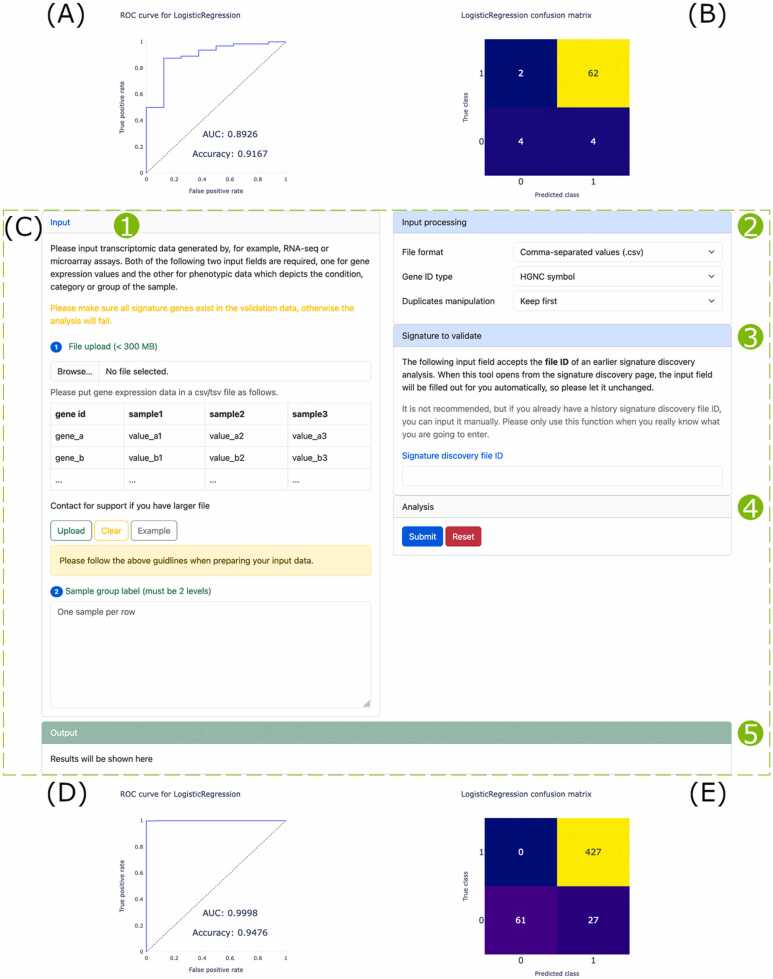
Screenshots and application of the ‘Signature discovery’ and ‘Signature validation’ tools. The layout of the web page of ‘Signature discovery’ is the same as that of ‘DE analysis’ so it is not shown here. (A-B) Model performance of the ecDNA gene signature discovered in the OV-2009 data evaluated by ROC curve (A) and confusion matrix (B). Signature discovery input data description: 20,373 genes × 237 samples. Signature discovery processing time: 82 s. Signature discovery non-default parameters: Set ‘Value type’ to ‘RNA-seq (other)’ and ‘ecDNA oncogene’ to ‘Yes’. (C) The web page of ‘Signature validation’ showing the input panel (1), input processing parameters panel (2), signature to validate panel (3), analysis controller panel (4), and output panel (5). (D-E) Validation of the discovered signature by ROC curve (D) and confusion matrix (E) in the OV-TCGA-GTEx data. Signature validation input data description: 58,581 genes × 515 samples. Signature validation processing time: 30 s.

For the application case, we used it to find an ecDNA gene signature capable of classifying samples into tumor and normal groups. We applied it on the OV-2009 data set and discovered a signature composed by four ecDNA genes (CDKN1A, BLM, FOXL2, and ALDH1A1). The best trained model is logistic regression that achieves an AUC and accuracy of 0.89 and 0.92, respectively (Fig. 7A), and the confusion matrix shows that most positives are correctly predicted as positive (Fig. 7B).

For further validation of the discovered ecDNA gene signature and the trained model on unseen data, the auxiliary ‘Signature validation’ tool was created (Fig. 7C). We designed it as an optional tool, so it is not accessible from the navigation bar. The ‘Signature validation’ tool is restricted to be implemented from ‘Signature discovery’ and becomes available following the completion of signature discovery. After uploading validation data (Fig. 7C1) and setting parameters (Fig. 7C2–3), the validation request can be submitted (Fig. 7C4). When the analysis finishes, results will be shown in the output box (Fig. 7C5).

Next, we applied ‘Signature validation’ on the OV-TCGA-GTEx data to validate the ecDNA gene signature and the trained model discovered in the OV-2009 data set. The ROC evaluation shows an AUC of nearly 1 and an accuracy of 0.95 (Fig. 7D), and the confusion matrix shows high levels of true positives as well (Fig. 7E).

## 4 Discussion

The ecDNA gene resource in ECGA provides a panorama of ecDNA genes in cancer. With annotation data and expression profiles integrated from external sources, it is a practical and useful start point for experimental oncologists to form novel hypotheses. Xena browser and GeneRanger are two popular places that provide uniform and standardized data about gene and protein expression across a variety of human cells and tissues from several large-scale projects, hence gene expression profiles from them were integrated into ECGA [29], [30]. This not only simplifies the visualization, access, and reuse of the huge volume of data in public atlases, but also enriches the data content in ECGA.

Due to ecDNA’s importance in oncogene amplification, intratumoral heterogeneity, and patient outcomes, we have seen the emergence of database resources for ecDNA in recent years. They provide high-quality data of ecDNA and borne genes, but they do not offer convenient and sophisticated ecDNA gene analysis which is only available in ECGA to date. We must acknowledge that ecDNA data is not explicitly offered in ECGA. They are only accessible via ecDNA gene browsing, unlike other databases where ecDNA information is easy to search, browse, and download.

Abnormally expressed genes in disease can explain disease mechanisms and may serve as drug targets for therapeutic intervention [40]. TargetRanger enables to identify genes and/or proteins that are aberrantly and specifically expressed only in the disease by comparing their reference expression levels across normal cells and tissues [29]. The critical role of ecDNA in cancer has been well known, and ecDNA genes can be hijacked and contribute to tumorigenesis. To identify highly expressed ecDNA genes in cancer compared to their expression in normal, the ‘Target discovery’ tool was created based on TargetRanger. Behind the scenes, abnormal expression is detected by two-tailed Welch’s *t*-test.

Venn diagram is a straightforward and intuitive way to show all possible logical relations between different sets. Taken a gene list derived from an experiment and ecDNA genes in ECGA as two sets, finding ecDNA genes in a candidate gene list is likely to resolve the problem of set relationships. Therefore, we created the ‘Venn analysis’ tool to detect the existence and analyze the proportion of ecDNAs in a gene list being studied by the user. To minimize text overlapping in the Venn diagram, both area-proportional and unscaled Venn diagrams are provided in the output.

In high-throughput gene expression studies, differential expression analysis and subsequent functional enrichment analysis have been integral parts in the data analysis workflow [41]. We developed the ‘DE analysis’ and ‘Enrichment analysis’ tools to simplify differential expression and pathway analyses tailored for ecDNA gene research. We integrated limma and DESeq2, so it is suitable for both microarray and RNA-seq data. The ecDNA gene resource forms a predefined collection of gene sets to perform enrichment analysis. Nowadays interactive plot for ORA-based enrichment analysis has been available in some web applications like g:Profiler and Enrichr [42], [43]. Our previous work pioneered the development of interactive visualizations for GSEA-based analyses [31]. Here interactive GSEA plot is available as well. The interactive feature will provide a better way for result exploration and interpretation.

Nowadays, ecDNA has been an emerging hallmark in human cancer, and oncogene amplification by ecDNA drives tumor evolution, drug resistance, and poor outcomes for patients across multiple cancers [14], [44]. An ecDNA gene signature encompassing nine genes has been successfully used to build a prognosis model in ovary cancer, highlighting its clinical potential [38]. Therefore, we developed the ‘Signature discovery’ tool to help users construct ecDNA gene signatures and build and evaluate clinical predictive models. In machine learning, although cross validation in train set and test set based on a single data set is a reasonable evaluation of estimators when obtaining independent data from third parties is difficult, it is better to validate trained models on unseen data. We also developed the ‘Signature validation’ tool to assist optional validation of a discovered signature model on a new data set when it is available.

To streamline data preprocessing, data transformation, feature encoding, feature selection, model train, model test, and model evaluation, we took the advantage of automated machine learning which wraps all these steps into a single pipeline. This enables ‘Signature discovery’ to construct and evaluate candidate ecDNA signatures across various machine learning models. It not only exponentially speeds up the experiment cycle but also help users to quickly find the best ecDNA signature to consider in the next step in their study. During signature analysis, differentially expressed genes are determined and used to reduce the feature space. The performance of all estimators available in the model library (Supplementary Table 3) are evaluated using cross-validation, and the output delivers a scoring grid of average cross-validated scores.

## 5 Conclusion

In the present study, we developed ECGA to explore ecDNA gene in cancer and perform ecDNA gene analyses. Though ecDNA gene information is also available in existing databases, the dedicated ecDNA gene analysis tools are only available in ECGA so far. We must acknowledge that although we have tried our best to generate a comprehensive catalogue of ecDNA genes in human cancers, we are limited by the scope of publicly available WGS data. In the future, we consider including more data types (such as ATAC-seq, 4C-seq, and Circle-Seq), analysis algorithms, and cancer samples. To sum up, ECGA is a centralized resource for ecDNA genes identified in cancers in a systematic manner and is a web-based application suite that covers a wide range of analysis requirements in the research of ecDNA gene in cancer. It will aid in better understanding of the molecular mechanisms of malignant tumors and facilitate the discovery of new therapeutic targets and biomarkers, thereby offering new perspectives for advancing cancer research and clinical practice.

## CRediT authorship contribution statement

**Li Peng:** Writing – original draft, Resources, Investigation, Funding acquisition. **Nan Zhou:** Writing – original draft, Visualization, Software, Methodology, Formal analysis, Data curation. **Xiaoqing Yuan:** Writing – review & editing, Resources, Project administration, Funding acquisition, Conceptualization. **Yuping Ning:** Writing – review & editing, Supervision, Funding acquisition, Conceptualization. **Jinku Bao:** Writing – review & editing, Funding acquisition, Conceptualization. **Huiran Sun:** Data curation. **Qiqi Luo:** Validation, Formal analysis. **Zhiyu Zhang:** Visualization, Validation, Data curation.

## Conflict of Interest

The authors have declared no conflict of interest.
