# Supplementary material for: ECGA: A web server to explore and analyze extrachromosomal gene in cancer
Source: Comput Struct Biotechnol J. 2024 Nov 5;23:3955–66. doi: 10.1016/j.csbj.2024.11.009 (PMC11584521; doi:10.1016/j.csbj.2024.11.009)
Supplement: Supplementary file 1 — Supplementary material [file mmc1.docx]

Supplementary Table 1. Parameters of all tools.

| Tool | Parameter | Value type | Default value | Description |
| --- | --- | --- | --- | --- |
| Shared parameters | | | | |
|  | Tissue | String | All | Select ecDNA genes of all tissues. |
|  | Disease | String | All | Select ecDNA genes of all diseases. |
|  | EcDNA oncogene | String | No | Select ecDNA gene, or ecDNA oncogene otherwise. |
|  | Ave. ecDNA gene score | Number | 0 | Select ecDNA genes with an average score of 0 or greater. |
|  | ecDNA hits | Number | 0 | Select ecDNA genes located on 0 or more ecDNAs. |
|  | ecDNA copy number | Number | 0 | Select ecDNA genes located on ecDNAs with a copy number of 0 or greater. |
|  | ecDNA size (bp) | Number | [0, 3e9] | Select ecDNA genes located on ecDNAs of a size between 0 and 3e9 base pairs. |
| Tool specific parameters | | | | |
| Venn analysis | Gene input | String or file | None | User’s gene list (one-column). |
| Enrichment analysis | Input (user gene list) | String or file | None | User's gene list (ranked or unranked, one-column or two-column). |
|  | Method | String | ORA | Method to perform enrichment analysis. |
|  | Background | String | None | Background gene for enrichment analysis. Available when “Method” is ORA. |
|  | Min gene set size | Number | 5 | The minimum size of a gene set to be considered. Available when “Method” is GSEA. |
|  | Max gene set size | Number | 1000 | The maximum size of a gene set to be considered. Available when “Method” is GSEA. |
|  | # permutation | Number | 1000 | The number of permutations to perform in assessing statistical significance. Available when “Method” is GSEA. |
|  | ecDNA gene score | Number | 0 | Select ecDNA genes with a score of 0 or greater. |
| Target discovery | Input (RNA-seq count) | File | None | User’s RNA-seq data set. |
|  | File format | String | .csv | File format. |
|  | Background | String | ARCHS4 | Normal background to compare with. |
| DE analysis | Input data source | String | File upload | Upload user’s data file or use pre-processed GDC TCGA data. |
|  | File upload | File | None | Upload user’s data file. Available when “Data source” is “File upload”. |
|  | Sample group label | String | None | Grouping labels for samples in the user’s data set. Available when “Data source” is “File upload”. |
|  | File format | String | .csv | File format. |
|  | Value type | String | RNA-seq (raw count) | The type of values of the data. |
|  | Gene ID type | String | HGNC symbol | The type of gene identifiers. |
|  | Duplicates manipulation | String | Keep first | Method to handle gene duplicates. |
|  | Fold change | Number | 2 | Minimal fold change to detect differentially expressed genes. |
|  | P value | Number | 0.05 | P of significance to be differentially expressed genes. |
| Signature discovery | File ID | String | None | Job ID to retrieve history results (within 48 hours). |
|  | Other | None | None | Shared parameters with DE analysis. |
| Signature validation | Signature discovery file ID | String | None | File ID of the corresponding signature discovery analysis. |
|  | Other | None | None | Shared parameters with DE analysis. |
