# Supplementary material for: ECGA: A web server to explore and analyze extrachromosomal gene in cancer
Source: Comput Struct Biotechnol J. 2024 Nov 5;23:3955–66. doi: 10.1016/j.csbj.2024.11.009 (PMC11584521; doi:10.1016/j.csbj.2024.11.009)
Supplement: Supplementary file 3 — Supplementary material [file mmc3.docx]

Supplementary Table 3. All available models.

| ID | Name | Estimator | Turbo |
| --- | --- | --- | --- |
| lr | Logistic Regression | sklearn.linear_model._logistic.LogisticRegression | TRUE |
| knn | K Neighbors Classifier | sklearn.neighbors._classification.KNeighborsClassifier | TRUE |
| nb | Naive Bayes | sklearn.naive_bayes.GaussianNB | TRUE |
| dt | Decision Tree Classifier | sklearn.tree._classes.DecisionTreeClassifier | TRUE |
| svm | SVM - Linear Kernel | sklearn.linear_model._stochastic_gradient.SGDClassifier | TRUE |
| rbfsvm | SVM - Radial Kernel | sklearn.svm._classes.SVC | FALSE |
| gpc | Gaussian Process Classifier | sklearn.gaussian_process._gpc.GaussianProcessClassifier | FALSE |
| mlp | MLP Classifier | sklearn.neural_network._multilayer_perceptron.MLPClassifier | FALSE |
| ridge | Ridge Classifier | sklearn.linear_model._ridge.RidgeClassifier | TRUE |
| rf | Random Forest Classifier | sklearn.ensemble._forest.RandomForestClassifier | TRUE |
| qda | Quadratic Discriminant Analysis | sklearn.discriminant_analysis.QuadraticDiscriminantAnalysis | TRUE |
| ada | Ada Boost Classifier | sklearn.ensemble._weight_boosting.AdaBoostClassifier | TRUE |
| gbc | Gradient Boosting Classifier | sklearn.ensemble._gb.GradientBoostingClassifier | TRUE |
| lda | Linear Discriminant Analysis | sklearn.discriminant_analysis.LinearDiscriminantAnalysis | TRUE |
| et | Extra Trees Classifier | sklearn.ensemble._forest.ExtraTreesClassifier | TRUE |
| xgboost | Extreme Gradient Boosting | xgboost.sklearn.XGBClassifier | TRUE |
| lightgbm | Light Gradient Boosting Machine | lightgbm.sklearn.LGBMClassifier | TRUE |
| dummy | Dummy Classifier | sklearn.dummy.DummyClassifier | TRUE |
